# Exploring the bi‐directional relationship between autophagy and Alzheimer’s disease

**DOI:** 10.1111/cns.13216

**Published:** 2019-09-10

**Authors:** Huang Kuang, Cheng‐Yong Tan, Hui‐Zhen Tian, Li‐Hua Liu, Mei‐Wen Yang, Fen‐Fang Hong, Shu‐Long Yang

**Affiliations:** ^1^ Department of Physiology, College of Medicine Nanchang University Nanchang China; ^2^ Department of Nurse Nanchang University Hospital Nanchang China; ^3^ Department of Experimental Teaching Center Nanchang University Nanchang China

**Keywords:** Alzheimer's disease, autophagy, genes and proteins, Tau, β‐amyloid

## Abstract

Alzheimer's disease (AD) is characterized by β‐amyloid (Aβ) deposition and Tau phosphorylation, in which its pathogenesis has not been cleared so far. The metabolism of Aβ and Tau is critically affected by the autophagy. Abnormal autophagy is thought to be involved in the pathogenesis of AD, regulating autophagy may become a new strategy for AD treatment. In the early stage of AD, the presence of Aβ and Tau can induce autophagy to promote their clearance by means of mTOR‐dependent and independent manners. As AD progress, the autophagy goes aberrant. As a result, Aβ and Tau generate continually, which aggravates both autophagy dysfunction and AD. Besides, several related genes and proteins of AD can also adapt autophagy to make an effect on the AD development. There seems to be a bi‐directional relationship between AD pathology and autophagy. At present, this article reviews this relationship from these aspects: (a) the signaling pathways of regulating autophagy; (b) the relationships between the autophagy and the processing of Aβ; (c) Aβ and Tau cause autophagy dysfunction; (d) normal autophagy promotes the clearance of Aβ and Tau; (e) the relationships between the autophagy and both genes and proteins related to AD: TFEB, miRNAs, Beclin‐1, Presenilin, and Nrf2; and (f) the small molecules regulating autophagy on AD therapy. All of the above may help to further elucidate the pathogenesis of AD and provide a theoretical basis for clinical treatment of AD.

## 1 INTRODUCTION

Alzheimer's disease (AD) is an age‐related neurodegenerative disease, which is the most prevalent form of senile dementia in the world.^1^ Clinically, it is characterized by progressive and irreversible cognitive dysfunction.^2^ The main pathological features of AD are neurofibrillary tangles formed by phosphorylated Tau protein aggregates and senile plaques formed by deposition of β‐amyloid (Aβ) peptide, respectively.^3^ The autophagy plays an important role in clearing damaged cells or organelles and long‐lived protein aggregates.^4^, ^5^ Autophagy can be either nonselective, as is commonly referred to as macroautophagy, microautophagy, and chaperone‐mediated autophagy, or selective, including mitochondrial autophagy (mitophagy). Among them, the macroautophagy (refer as “autophagy” in this article) is widely studied and most relevant to AD. Moreover, recent studies have found that mitophagy defects are closely associated with AD development.^6^ Mitophagy is an autophagic process of selectively removing excess or damaged mitochondria, which is a kind of macroautophagy.^7^ Autophagic mechanism can be divided into five processes: (a) isolation membrane appears in cells; (b) the isolation membrane expands continuously under the action of related proteins, and surrounds the aging proteins, mitochondria, and other organelles around it; (c) the isolation membrane grows into an autophagosome with a double membrane structure; (d) the outer membrane of autophagosomes is fused with lysosomes; and (e) autophagy‐lysosomes are formed by degradation of membrane and inclusions into amino acids and other small molecules by hydrolytic enzymes.^5^

The relationships between the roles of autophagy and the pathogenesis of AD have received widespread concern. Autophagy is constitutively active and efficient in normal neurons, while the autophagy dysfunction is observed in AD.^8^ The regulation of autophagy involves in complex signaling transduction pathways, which can be mainly divided into two aspects: the mTOR‐dependent manner and mTOR‐independent manner; however, both the two regulating pathways were found to be abnormal in AD.^9^, ^10^ There may be a bi‐directional relationship between autophagy dysfunction and AD pathology: Aβ and Tau, which constitutes a vicious cycle to worsen the AD. On the one hand, it has confirmed that induction of autophagy can promote the clearance and degradation of AD pathology in the brain of AD patients and animal model.^11^ On the other hand, the autophagy goes aberrant accompanied AD progression,^12^ and both the increased Aβ and Tau expression lead to defective autophagy and mitophagy in AD.^13^ In return, it has reported that the autophagy‐lysosomal pathway also plays a role in secretion of Aβ and Tau,^14^, ^15^, ^16^ which further deteriorate autophagy function and accelerate AD development. Besides, some genes and proteins, such as transcription factors EB (TFEB), miRNAs, Beclin‐1, Presenilin, and Nrf2, and so on, which are crucial for the regulation of autophagy, may be closely related to the pathogenesis of AD. While the levels of their expression in AD is dysregulated, which are associated with both autophagy and AD pathology, may directly or indirectly influence the relationship between autophagy and metabolism of AD pathology. Given that, the roles of autophagy in AD is to be stated as follows.

## 2 REGULATION OF AUTOPHAGY

### 2.1 Regulating autophagy via mTOR‐dependent pathway

**Figure 1:**
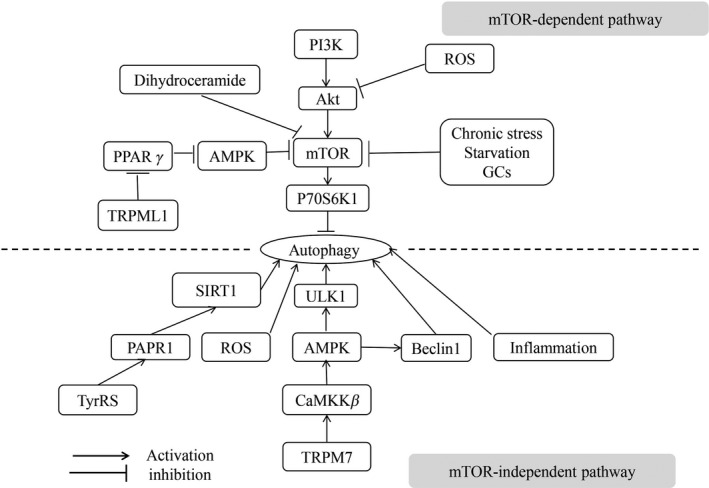
The signaling transduction pathways of regulating autophagy. The autophagy can be regulated by two pathways: mTOR‐dependent pathway and mTOR‐independent pathway. The mTOR is the core molecule in regulating autophagy, and phosphorylation of mTOR can lead to the phosphorylation of P70S6K1 which is a subtrate protein of mTOR, thereby inhibiting autophagy. In a physiological situation, the autophagy can be activated by some factors such as chronic stress, starvation, and GCs via inhibiting the expression of mTOR. The PI3K/Akt/mTOR and TRPML1/PPARγ/AMPK/mTOR are negnative and positive pathways in regulating autophagy respectively, inhibition and activation of the two pathways can activate autophagy. Besides, the Dihydroceramide is a newly positive regulator of autophagy via mTOR. ROS induces autophagy through both mTOR‐dependent and independent pathways. The inflammatory activation of microglia also plays a role in activating autophagy. Others, the TyrRS/PARP1/SIRT1 and TRPM7/CaMKKβ/AMPK are two positive pathways in regulating autophagy

The mammalian target of rapamycin (mTOR) was an important serine‐threonine protein kinase, which consisted of two complexes: the mTOR complex 1 (mTORC1) and mTOR complex 2 (mTORC2). The mTOR was a classical regulator of autophagy, in which its activity was regulated by some factors such as chronic stress, starvation, and glucocorticoids.^17^, ^18^ According to many in the field, the autophagy can be regulated by the mTOR‐dependent pathway, while this pathway was found to be activated in patients with AD in early stage.^19^, ^20^ The phosphoinositide 3‐kinases (PI3K) and protein kinase B (Akt/PKB) were two upstream signaling molecules of mTOR respectively, and the two molecules together with mTOR constituted the PI3K/Akt/mTOR pathway, which involved in autophagy regulation; inhibition, or blockage of any molecule of this pathway exerted the biological effect on promoting autophagy, following accelerating the clearance of Aβ in AD.^21^, ^22^, ^23^ The Akt was a positive regulatory kinase upstream of mTOR that increased mTOR activity by direct or indirect phosphorylation of mTOR, leading to the phosphorylation of mTOR downstream substrate protein, p70S6K1, then inhibiting autophagy initiation.^24^ In addition, adenosine 5'‐monophosphate‐activated protein kinase (AMPK), a vital molecule that triggered autophagy, was also located in upstream of the mTOR pathway, together with the peroxisome proliferator‐activated receptors‐γ (PPAR γ), and mTOR constituted the PPARγ/AMPK/mTOR pathway for regulating autophagy.^25^ Dihydroceramide was previously considered to be a regulator of autophagy, while the underlying mechanism is unclear. A recent study has found that Dihydroceramide desaturase 1, an enzyme that catalyzed the generation of Dihydroceramide, which can downregulate the levels of mTORC1,^25^ and in turn inhibited the p70S6K1 activity and promoted autophagy eventually. The findings of this study suggested that the Dihydroceramide was not the real autophagy regulator, instead, the Dihydroceramide desaturase 1 can regulate autophagy through autophagy. In addition, the transient receptor potential Mucolipin‐1 (TRPML1) was also considered to be one of the autophagy regulators, and it was found that the TRPML1 regulated autophagy via PPARγ/AMPK/mTOR pathway.^26^ Besides, the reactive oxygen species (ROS) was also likely to regulate autophagy via the Akt/mTOR pathway (Figure 1).^27^

### 2.2 Regulating autophagy via mTOR‐independent pathway

(a) The TRPM7/CaMKKβ/AMPK pathway. Increased phosphorylated form of AMPK activated autophagy. The upstream regulator of AMPK was Ca^2+^‐dependent protein kinase kinase β (CaMKKβ), which was regulated by Ca^2+^ influx. It was reported that the Ca^2+^ influx through transient receptor potential melastatin (TRPM7) regulated basal autophagy via CaMKKβ/AMPK pathway and downregulated the endogenous TRPM7 channel decreasing the level of basal autophagy.^28^, ^29^ In more detail, the AMPK was an upstream signaling molecule that directly phosphorylated the serine 317th and 777th sites of the autophagy initiation kinase, UIK1, and then regulated autophagy.^30^ Besides, AMPK was also an upstream signaling constituting Beclin‐1‐related proautophagy complex, and AMPK directly phosphorylated the serine 91th/94th in Beclin‐1 activating autophagy.^31^ (b) The inflammation‐mediated pathway. Inflammatory activation of microglia enhanced the transportation of p‐Tau in neurons and promoted the degradation of p‐Tau in lysosomes.^32^ This process was related to the increased autophagic flux in microglia, it could conceivably be hypothesized that mild inflammation of neurons was an important condition for autophagic flux of neurons activation. (c) The ROS‐mediated pathway. The accumulation of ROS in neuronal cells was regarded as another characteristic of AD progression.^33^ In the context of ROS oxidation, proteins can be polymerized, as a result of forming the protein aggravates.^34^ ROS was able to induce autophagy via autophagy‐related gene 4 (Atg4) and blocked the Atg4 expression can significantly downregulate the levels of Beclin‐1 and light chain 3‐II (LC3‐II) protein expression (two proteins of autophagy markers).^27^ (d) The TyrRS/PARP1/SIRT1 pathway. The activation of Tyrosyl transfexr‐RNA synthetase (TyrRS) can stimulate PARP1 and ultimately led to the activation of SIRT1, which was a positive regulator of basal autophagy, and it can mediate autophagy through regulating the expression of several autophagy‐associated proteins (Figure 1).^35^

## 3 AUTOPHAGY AND Aβ

### 3.1 The relationships between the autophagy and the processing of Aβ

The β‐Site amyloid precursor protein (APP) was a type I trans‐membrane protein, which sequentially cleaved by β‐secretase (BACE1) and γ‐secretase to produce Aβ.^36^ Increased BACE1 and γ‐secretase activities were described to promote the APP converted to Aβ, thus accelerating AD progression.^37^ In the processing of APP, the autophagy played a crucial role. The phenomenon observed in AD animal models was the activation of Atg5‐dependent autophagy can promote the degradation of APP early, thus preventing the accumulation of Aβ.^38^ Sirtuin1 (SIRT1), as a positively related molecule of autophagy, in which its activation can upregulate the expressions of Beclin‐1, Atg5, and LC3‐II; then caused the APP‐CTFβ levels and Aβ clearance rate decreased and accelerated, respectively.^39^ The APP/PS1 transgenic mouse models have been proverbially used in the past and current to investigate the mechanism of AD, study carried out by using this model as experimental object, found that inhibiting the mTOR pathway to promote autophagy can reduce the levels of BACE1 expression.^40^ The PPARα regulated autophagy in the nervous system, and PPARα‐mediated autophagy affected AD. Recent studies have confirmed that the activation of PPARα decreased Aβ pathology and reversed memory deficits by promoting the clearance of APP via activating autophagy in APP/PS1 mice. A reduced level of Aβ in hippocampus and cortex tissues after treatment with PPARα agonists was observed, which promoted the recruitment of microglia and astrocytes to the vicinity of Aβ plaques and enhanced autophagosome biogenesis.^41^ These results indicated that PPARα was a crucial factor regulating autophagy in the processing of Aβ. The mutation of APP can also cause abnormal autophagy and worsen AD progression.^42^ The expression of mutant APP was reported to be associated with impaired mitochondrial energy metabolism in AD neurons,^43^ and it has been found that the hippocampal mutant APP caused the mitophagy dysfunction in mAPP hippocampal cells and 12‐month‐old APP transgenic mice.^42^, ^44^ At the same time, autophagy failure can activate γ‐secretase complex to promote APP production and cause Aβ production.^45^ This was a malignant loop that can exacerbate AD. Besides, the autophagy inhibitor 3‐Methyladenine (3‐MA) can upregulate the γ‐secretase components to increase its activity and promote production and accumulation of Aβ.^46^ However, the abnormal activation of autophagy can also promote APP cleavage, contributing to Aβ production.^47^, ^48^ The above experimental results indicate that APP is a substrate for autophagy in the early stage of Aβ production, and normal autophagy function is essential for the clearance of APP. However, how APP became a substrate for autophagy still needed further clarification.

### 3.2 The relationships between the abnormal autophagy and Aβ

In the early stage of AD, the Aβ formation can activate the autophagy,^49^ and then, the Aβ can be degraded by transporting from autophagosome to lysosome.^50^ However, the autophagy became aberrant and the Aβ clearance cannot be performed normally as AD progression.^47^, ^51^ And the activation of autophagy did not have beneficial effects on AD pathology and cognitive deficit.^21^, ^52^, ^53^, ^54^, ^55^ Studies have shown that Aβ_1‐42_ was localized in dysfunctional autophagic vesicles in Drosophila expressing Aβ_1‐42_, and this vesicle may be a source of extracellular Aβ plaque accumulation.^56^ Still, evidence suggested that the autophagy can participate in Aβ secretion through the secretory pathway from the endoplasmic reticulum to the Golgi apparatus to the plasma membrane or the secretory lysosomal pathway; moreover, the lack of neuronal autophagy will attenuate Aβ secretion.^14^, ^15^ This observation may support the hypothesis that autophagy played a dual role in Aβ degradation and secretion, so further study of the dual role of autophagy in Aβ clearance and secretion may contribute to better understand the pathogenesis of AD.

It has been suggested that the persistent accumulation of Aβ in AD late stage induced aberrant autophagy,^42^ which caused neuronal dysfunction and further exacerbatd AD symptoms.^37^ In addition, the Aβ‐derived diffusible ligands (ADLLs), one of the Aβ toxic forms, which were involved in AD development through regulating autophagy.^57^ As ADLLs were exposed to neuronal cells, the phosphorylated p70S6K1 expression levels were significantly reduced, suggesting that the inhibition of mTOR pathway involved in ADLLs‐induced abnormal autophagy.^57^ Besides, Aβ can upregulate the expression of NADPH oxidase4 (NOX4) to increase ROS aggregation, following autophagy over‐activation, which caused neuronal cell death; while the inhibition of NOX4 expression and reduction in ROS levels can prevent autophagy from over‐activation and protect neuronal cells from death.^58^ Receptor of advanced glycation end‐products (RAGE) was a key receptor in mediating Aβ toxicity, and it confirmed that Aβ_1‐42_ oligomers can induce aberrant autophagy via the RAGE‐mediated pathway, thus disrupting the tight junction protein in blood‐brain barrier, which can worsen the progression of AD.^59^ Aβ can also result in autophagic dysfunction. It was found that autophagy dysfunction occurred in astrocytes after treatment with Aβ, following p62 and LC3‐II/LC3‐I conversion rate aggregated and decreased, respectively.^60^

Mitochondrial dysfunction, damaged mitochondria, and autophagy have been extensively reported in patients with AD.^6^ These mitochondrial abnormalities may be due to the interaction of Aβ with voltage‐dependent anion channel 1 protein (VDAC1) and dynamin‐related protein 1 (Drp1). Increased production of Aβ and the interaction of Aβ with VDAC1 and Drp1 are critical factors in abnormal mithphagy, mitochondrial dynamics, and synaptic damage.^61^, ^62^ PTEN‐induced putative kinase 1 (PINK1) is crucial to the maintenance of mitochondrial function by promoting the removal of damaged mitochondria via mitophagy.^63^ Studies have found the decreased levels of PINK1 were associated with Aβ pathology, and PINK1‐dependent Aβ pathology through mitophagy contributing to the synaptic and cognitive dysfunction in the pathogenesis of AD.^64^ However, PINK1 overexpression promoted the clearance of damaged mitochondria by promoting mitophagy signaling via activation of autophagy receptors (OPTN and NDP52), alleviating Aβ‐induced loss of synapses and cognitive decline in AD.^64^ Moreover, the hippocampal Aβ could lead to the decreased PINK1 expression to inhibited mitophagy and cause cognitive decline in a mouse model of AD.^44^

### 3.3 The relationships between the normal autophagy and Aβ

Normal autophagy activation or enhancement can effectively eliminate Aβ aggregates and inhibit Aβ‐induced neurodegeneration in the early stage of AD. The activity of SH‐SY5Y cells can be inhibited after treatment of Aβ_1‐42_, while the autophagy inducer rapamycin, which was applied to activate autophagy showed decreased Aβ_1‐42_ levels, and the harmful effects such as cytotoxicity induced by Aβ_1‐42_ simultaneously alleviated.^65^, ^66^ Moreover, increasing the P62 expression to activate autophagy through the mTOR‐dependent pathway in the brain of APP/PS1 AD mouse model, which can exert multiple beneficial effects: reduced Aβ levels, ameliorated senile plaque burden, and decreased cognitive deficit.^67^ However, after the APP/PS1 AD mouse model was administered with rAAV/Aβ oral vaccination, the proportion of LC3B‐II/LC3B‐I in the brain was upregulated, indicating an enhancement in autophagy, but this accompanied decreased P62 expression.^68^ The result was contrary to the previous experimental results,^67^ and suggested that the role of P62 in mediating autophagy and Aβ clearance remaining to be elucidated. Also, the over‐activation of gene expression in mTOR signaling pathway may serve as a disruption, which was related to AD development. It confirmed the AD mouse model with gene knockout can inhibit the mTOR‐dependent pathway, thus inducing autophagy, which in turn reduced Aβ deposition and rescues memory deficit.^69^ This can provide a theoretical basis for the development of anti‐AD drugs based on the mTOR pathway. The autophagic flux recovery was crucial for reversing the spatial learning and cognitive deficit caused by Aβ.^70^ Increased Aβ level in AD was thought to be associated with decrease of the release of insulin‐degrading enzyme (IDE) in aging microglia, and IDE was secreted to extracellular, and it can degrade extracellular Aβ, and this was a process that relied on autophagic flux, and recovery of autophagic flux can increase the secretion of IDE and promote enzymatic hydrolysis of Aβ.^71^, ^72^ Moreover, it was reported that cerebral Aβ burden may impair insulin signaling via promoting autophagy‐lysosomal degradation of insulin receptors and low‐density lipoprotein receptor‐related protein‐1, thereby contributing to impaired cerebral insulin effects.^73^ However, excessive autophagic flux may likewise add to the accumulation of LC3‐II and autophagosome, which affects the clearance of Aβ.^74^

The aggregation of Aβ was able to interfere with Ca^2+^ homeostasis and caused mitochondrial dysfunction, which was firmly related to the AD pathogenesis.^75^ The moderate activation of autophagy can regulate Ca^2+^ homeostasis and maintain mitochondrial membrane potential to alleviate Aβ_1‐42_‐induced cytotoxicity.^75^ Besides, the level of 12/15‐lipoxygenase (12/15‐LO) was upregulated in the brain of AD patients, and its expression level affected the AD progression.^76^ The Aβ level was significantly reduced in the AD mouse model after treatment with the 12/15‐LO inhibitor, and further studies revealed this effect was associated with the 12/15‐LO inhibitor can activate the autophagy.^76^ Alborixin, an ionophore, as an autophagy inducer, was found that significantly cleared Aβ in microglia and primary neuronal cells by inducing autophagy. Induction of autophagy was accompanied by up‐regulation of autophagy proteins Beclin‐1, Atg5, Atg7, and increased lysosomal activities. Autophagy induced by alborixin was associated with inhibition of the PI3K/Akt pathway.^77^ Flat movement has been reported can significantly reduce the area and load of Aβ plaques in the APP/PS1 AD mouse model and improve cognitive deficits in AD mice,^22^ which involved in regulating autophagy activity, and enhanced autophagy activity was associated with the inhibition of PI3K/Akt/mTOR pathway.^22^, ^78^ In addition, enhanced autophagy can reduce oxidative stress and apoptosis in hippocampus, reducing the deposition of Aβ and thus improving the neurological dysfunction caused by Aβ.^79^ These results revealed the intervention of autophagy can reverse the toxic effects of Aβ and improve AD symptoms. However, the autophagy regulatory pathway is complex, how to screen out the most appropriate intervention still needs further research, and the mTOR pathway may be a suitable candidate pathway.

Interestingly, the ability of autophagy to clear Aβ may differ in genders. Epidemiological survey showed that women have a higher incidence of AD than men, and this difference may be related to autophagy. This conjecture was supported by these evidences, which the cells contained two X chromosomes expressing lower levels of autophagy‐related proteins, and both estrogen and progesterone produced by women can inhibit the level of basal autophagy, and the lower level of basal autophagy may impair the ability of neurons and microglia to clear Aβ.^80^ While other studies have shown that ovarian hormones instead can enhance the autophagy and promote the clearance of Aβ.^60^, ^81^ Therefore, further studies were needed to explore the role of ovarian hormones in mediating the relationship between autophagy and AD.

## 4 AUTOPHAGY AND TAU

### 4.1 The relationships between the abnormal autophagy and Tau

Phosphorylation of Tau was another pathological feature of AD. Although the ubiquitin‐proteasome system (UPS) was considered to be the main pathway for degradation of Tau, the autophagy may be another effective way to degrade.^82^, ^83^ In addition, phosphorylated Tau may also cause abnormal autophagy.^84^ Studies have shown that dysfunction of the autophagy‐lysosomal system led to the formation of Tau oligomers, and this was the first direct evidence that autophagy dysfunction involved in Tau aggregation.^85^ MiR‐132/212 targeted Tau mRNA to regulate Tau expression, and the downregulation of miR‐132/212 expression in the brian of AD patients led to Tau aggregation, and the role of miR‐132/212 in regulating Tau aggregation was found to be associated with autophagy dysfunction.^86^ POLDIP2 was a DNA polymerase δ interacting protein, and it was also a regulatory molecule for Tau aggregation; it confirmed the overexpression of POLDIP2 can inhibit autophagy, thus inducing Tau aggregation eventually.^87^ Besides, as a phosphorylated Tau‐autophagy receptor, the autophagy adaptor protein 52 (NDP52) can promote the elimination of phosphorylated Tau through autophagy.^88^, ^89^ However, the amount of autophagic vesicles (AV) containing NDP52 in the cortex and hippocampus of AD model was significantly increased, and the expression level of NDP52 protein and phosphorylated Tau and LC3‐II were also correspondingly upregulated, suggesting that autophagy was dysfunctional in AD model mice.^89^ The above studies suggested that damage to autophagy activity plays a key role in phosphorylated Tau aggregation. Notably, as Aβ we mentioned before,^14^, ^15^ Tau was also secreted via an autophagy‐mediated secretory pathway in neurons. It was reported that Tau secretion was promoted by autophagy inducers and downregulated by beclin‐1 knockdown or autophagy inhibitors derived from human wild type tau‐overexpressing SH‐SY5Y cells.^16^ Besides, the accumulation of hippocampal phosphorylated Tau is responsible for abnormal mitophagy function, mitochondrial dynamics hippocampal‐based learning and memory impairments in Tau mice.^90^ It has reported that the phosphorylated Tau can also interact with VDAC1 and Drp1, likely leading to mitochondrial dysfunction and abnormal mitophagy, ultimately possibly leading to neuronal damage and cognitive decline. 62, 91

### 4.2 The relationships between the normal autophagy and Tau

Normal autophagy was the main pathway for the removal of phosphorylated Tau in neurons, and autophagy activation or enhancement can effectively promote the clearance of Tau.^92^, ^93^ Both inhibition of the mTOR‐dependent pathway and the mTOR‐independent pathway can ameliorate Tau lesions in AD through inducing autophagy.^93^, ^94^, ^95^ It confirmed that selenium‐methionine (Se‐Met) activated autophagy through the AMPK‐mTOR pathway, and then promoted the clearance of Tau in neurons and improved cognitive ability of AD model mice.^96^ Decreased synaptic excitability is one of the earliest detectable changes in AD development.^97^ Inhibition of synaptic excitation will upregulate Tau oligomer levels, and oligomeric aggregates were in swollen lysosomes; while chronic synaptic stimulation elevated the autophagic flux, promoted the lysosomal degradation, reduced the Tau level, and recovered the lysosomal size.^97^ The autophagosome formation can also help to promote the clearance of Tau. The inhibitors that used to block the expression of cholesterol acyltransferasein in AD mice model, and it found that the autophagy was enhanced and the formation of autophagosome was induced respectively, which accompanied reduced phosphorylated Tau contents.^98^ Furthermore, blocking the 12/15 LO enzyme‐mediated pathway can also enhance autophagy and promote the clearance of Tau.^99^ Similarly, increased autophagic flux also decreased Tau aggregate levels.^100^ The AD mice that were treated with daily intra‐peritoneal injection of Pimozide revealed that Pimozide increased autophagic flux through the mTOR‐independent AMPK‐ULK1 axis, thus reducing soluble oligomers and NP40 insoluble aggregate levels of phosphorylated Tau in nerve cells and rescuing memory impairment.^100^ The autophagosome‐lysosome fusion and degradation required the formation of endosomal sorting complex required for transport (ESCRT) complex. ESCRT‐III, which contained IST1 (IST1 factor associated with ESCRT‐III) subunit, a positive modulator for the formation of ESCRT complex. ESCRT‐III subunits dysfunction resulted in autophagosome accumulation. The Tau accumulation inhibited IST1 expression and thus disrupted ESCRT‐III complex with decreased autophagosome‐lysosome fusion. However, up‐regulating IST1 in Tau transgenic mice attenuated autophagy deficit reduced Tau aggregation and ameliorated synaptic plasticity and cognitive decline.^101^

## 5 THE RELATIONSHIPS BETWEEN THE AUTOPHAGY AND BOTH GENES AND PROTEINS RELATED TO AD

### 5.1 Transcription factor EB

The transcription factor EB (TFEB) was a helical loop spiral transcription factor, in which a major regulator for lysosomal biogenesis. Promoting the nuclear translocation of TFEB in the cortex that upregulated the transcription of genes associated with autophagy and lysosome.^102^ Changes in expression of TFEB have been found to be evidently associated with abnormal autophagy in brain tissue of AD patients.^103^ The level of TFEB in the brain of AD patients was decreased accompanied abnormal autophagy, and overexpression of TFEB enhanced autophagy and improved autophagic flux in AD patients.^103^, ^104^, ^105^ The AMPK‐SIRT1‐TFEB pathway was recently reported to activate lysosomal function regulating autophagy in the brain, whether this pathway involved in clearance of Aβ and Tau needed further experiments.^102^ The TFEB was particularly relevant to the degradation of Tau, and it was effective in clearing Tau abnormal aggregates in Tau pathology mice.^106^ The TFEB overexpression reduced the levels of phosphorylated Tau in the cortex and hippocampus of AD mice and ameliorated the behavioral defect and neurodegeneration in AD mice.^107^ In addition, TFEB was also associated with Aβ clearance in brain tissue of AD patients.^105^ Overexpression of TFEB restored the autophagic flux blocked by Aβ_1‐42_ in AD model mice^108^; also enhanced the expression and activity of cathepsin D, which removed the lysosomal acidic environment interfered by Aβ_1‐42_, and promoted the fusion of autophagosome with lysosome. Aβ oligomers were also involved in regulating TFEB nuclear translocation and activating related genes that associated with autophagy function.^109^ In summary, the process of TFEB regulating autophagy was closely related to the pathogenesis of AD. Overexpression of TFEB promoted the removal of two major pathological features of AD: phosphorylation of Tau and Aβ and significantly improved the clinical symptoms of AD. Based on this, it can be speculated that further study about the role of TFEB in AD had a promising prospect for finding the underlying mechanism of AD and the anti‐AD drugs.

### 5.2 MicroRNA

The microRNAs (miRNAs) were small, noncoding single‐stranded RNAs. In recent years, some cases showed that miRNAs expression vary in brain tissue of AD patients. It were found to be relieved that the lesions of the AD when the miR‐124 was injected into the bilateral dentate gyrus of the hippocampus of the AD model mice; further studies showed that miR‐124 indirectly inhibited abnormal autophagy via BACE1‐regulated autophagy pathway, thus exerting its neuroprotective effects.^110^ MiR‐214‐3p was a negative regulator of autophagy in hippocampal neuron by directly and negatively targeting the 3´‐untranslated region of Atg12, and it was downregulated in AD patients and AD model mice. And the injection of miR‐214‐3p into the hippocampus ameliorated cognitive deficit.^111^ MiR‐299‐5p was also a potent autophagy regulator, and it inhibited neuronal abnormal autophagy both in vivo and in vitro, thus reducing hippocampal neuronal apoptosis improving the cognitive function in transgenic AD model mice. Mechanistically, Atg5 was verified as a direct target of miR‐299‐5p, and decreased Atg5 expression inhibited autophagy.^112^ Besides, recent studies have found that the autophagy also be regulated by miR‐101a via the MAPK pathway and might be a new mechanism in AD.^113^

**Figure 2:**
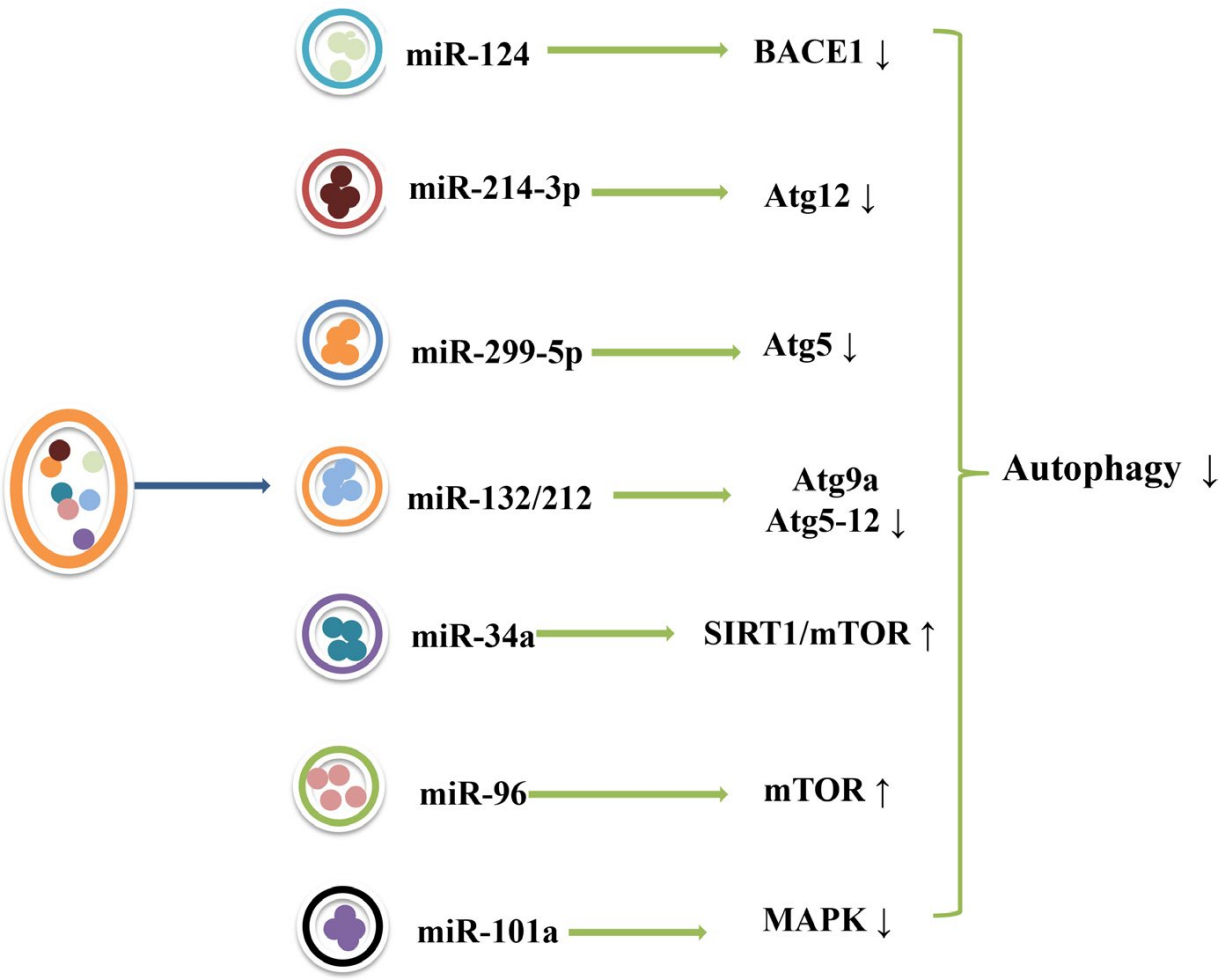
The related miRNAs and target genes/pathways that regulate autophagy. The miRNAs expression altered in AD patients and animal models. MiR‐124 inhibited abnormal autophagy via BACE1‐regulated autophagy pathway ameliorating AD pathology; miR‐214‐3p and miR‐299‐5p ameliorated cognitive deficit by negatively and respectively targeting the expression of Atg12 and Atg5 in AD mice; miR132/212 also associated with autophagy dysfunction by targeting the expression of Atg9a and Atg5‐12; miR‐34a regulated autophagy through SIRT1/mTOR pathway. The autophagy also be regulated by miR‐101a via the MAPK pathway. Finally, miR‐96 could regulate autophagy through the mTOR pathway to mediate the role of chronic cerebral hypoperfusion in the pathogenesis of AD

The miR‐132/212 were located on the bicistronic site on human chromosome 17 (mouse chromosome 11), which directly participated in endogenous Tau expression, phosphorylation, and aggregation. The levels of miR‐132/212 expression were downregulated in AD,^86^ which also associated with autophagy dysfunction by targeting the expression of Atg9a and Atg5‐12, but the specific role and relationship still need to be better studied. In addition, the expression of miR‐34a in AD brain was also observable, and miR‐34a was involved in autophagy regulation. After downregulating miR‐34a expression, autophagy can be activated via the SIRT1/mTOR pathway.^114^ Chronic cerebral hypoperfusion (CCH) is one of the high‐risk factors for AD, and miR‐96‐mediated mTOR‐dependent autophagy has been shown to be involved in its pathogenesis.^115^ Because, the miR‐96 levels were significantly elevated, and the amount of LC3 and the level of Beclin‐1 positive autophagosomes increased in the CCH model mice, while mTOR levels decreased. The above changes were reversed after injection of miR‐96 RNA antagonists, hinting that miR‐96 may regulate autophagy through the mTOR pathway to mediate the role of CCH in the pathogenesis of AD (Figure 2)^115^

### 5.3 Beclin‐1

The Beclin‐1 was an important protein that regulated the function of phagocytic receptor. Beclin‐1 has been found to regulate Aβ toxicity and neurodegenerative effects in animal models of AD. In recent years, it has confirmed that Beclin‐1 was impaired in AD, and the overall expression of Beclin‐1 was downregulated in AD’s brain.^116^, ^117^ A growing body of evidence suggested that Beclin‐1 played a significant role in autophagy, and the identification of Beclin‐1 protein modification has also been shown that Beclin‐1 involved in autophagy regulation, but the specific mechanism is unclear.^118^ In addition, Beclin‐1 can behave as Aβ regulator through autophagy. Aβ_1‐42_ induced Beclin‐1‐dependent autophagy in PC12 cells, and the expression of Beclin‐1 was positively correlated with cell viability.^119^ The lack of Beclin‐1 in neurons may cause Aβ peptide deposition,^120^, ^121^ and its overexpression reduced Aβ accumulation.^120^

### 5.4 Presenilin

Presenilin (PS), an intra‐membrane protease, including PS1 and PS2, both the two subtypes were mainly involved in neuronal Aβ information and contained a γ‐secretase complex catalytic site. Selective phosphorylation of PS1 at the serine 367th site can accelerate autophagosome‐lysosomal fusion and promote autophagy to downregulate Aβ expression.^122^, ^123^ The PS1 mutation was also one of the major causes for familial Alzheimer's disease (FAD). PS1 mutation aggravated autophagy and lysosomal lesions in AD patients, which were characterized by increased lysosome PH. Similarly, the loss of PS1 may lead to severe autophagy impairment in neuronal stem cells (NSCs), aggravating AD lesions, and its underlying mechanism was that deletion of PS1 inhibited the ERK/CREB signaling pathway and activated GSK3 to downregulate the TFEB expression in NSCs.^124^ Besides, recent studies have reported that PS2 mutation impaired autophagy by causing a block in the degradative flux at the level of the autophagosome‐lysosome fusion step. Importantly, FAD‐PS2 impaired autophagy was depended on its ability to partially deplete ER Ca^2+^ content, thereby attenuated cytosolic Ca^2+^ response upon IP3‐linked cell stimulations. These results indicate the significant role for Ca^2+^ signaling in regulating autophagy and reveal a novel mechanism by which FAD‐linked PS alters the autophagy process.^125^

### 5.5 Nrf2

Nuclear factor E2‐related factor 2 (Nrf2) was a key transcription factor against oxidative stress. Nrf2 can induce the expression of NDP52 and reduce phosphorylated Tau levels.^106^, ^126^ While, the knockdown of Nrf2 can observably increase the level of phosphorylated Tau.^127^ Currently, the Nrf2 was reported as a regulator of autophagy genes. Nrf2 was identified to regulate 9 autophagy genes and exhibited reduced expression of autophagy genes and more intracellular of Tau aggregates in Nrf2‐knockout mice. Also, the Nrf2‐regulated autophagy marker SQSTM1/P62 was reduced in the absence of Nrf2.^128^ Nrf2 upregulated the level of P62 and NDP52, which was receptor that promoted selective autophagy by simultaneously interacting with LC3 and cargo on autophagosome to maintain cell homeostasis. P62 was also more specific to regulate the Nrf2 expression, both of them formed a positive feedback loop to promote the clearance of Tau together by autophagy.^127^

### 5.6 Autophagy‐targeting small molecules and their implication on AD therapy

In recent years, AD has increasingly posed a threat to old people. With the development of drug structure and more thorough research on the molecular mechanism and related researches on AD, the research on AD related drugs has also made some progress. Early on, it was generally believed that the pathogenesis of AD was based on the cholinergic hypothesis, that is, insufficient acetylcholine was the cause of AD.^129^ Subsequently, it was found that drugs based on this design could only relieve AD. Currently, it is more likely that neuronal toxic proteins such as Aβ aggregation, Tau hyperphosphorylation, and abnormal or insufficient autophagy are the causes of AD.^7^, ^85^, ^130^ The complex autophagy process signal transduction includes many pathways, so the discovery and design of drugs regulating these pathways may be an important method for the treatment and prevention of AD. In this article, we briefly describe the recent findings of using small molecules (DNLA,^131^ EVOO,^132^ LANDO,^133^ SYK,^134^ ERβ,^135^ Ori,^136^ and TMED10^137^) to regulate autophagy and their implication on AD therapy (Table 1).

**Table 1:** Recent findings of using small molecules to regulate autophagy and their implication on AD therapy

Small molecules	Targeting autophagy machinery	Effect on AD	References
DNLA	Increases autophagic flux	Attenuates axonal degeneration of hippocampus	131
EVOO	Activates AMPK‐ULK1 pathway	Attenuates neuroinflammation	132
LANDO	Increases LC3‐II degradation	Promotes Aβ clearance and attenuates cognitive deficits	133
SYK	Inhibits mTOR pathway	Attenuates Tau accumulation, neuronal and synaptic loss	134
ERβ	Increases LC3‐II degradation and interacts with Atg7	Promotes Tau degradation and neuroprotective effect	135
Ori	Increases LC3‐II, P62 and cathepsin D degradation	Promotes learning and memory and Aβ clearance	136
TMED10	Activates Atg4B	Attenuates Aβ production	137

Abbreviations: DNLA, Dendrobium nobile Lindl alkaloid; ERβ, estrogen receptor β; EVOO, extra‐Virgin Olive Oil; LANDO, LC3‐associated endocytosis; Ori, orientin; SYK, spleen tyrosine kinase.

## 6 CONCLUSION AND PROSPECT

Although the specific pathogenesis of AD has not yet been elucidated, a growing number of studies point to the catabolic process of autophagy, and it is found to play a protective and beneficial role in the pathogenesis of early AD, while it is dysfunctional and aberrant with AD progressing, thus aggravating AD symptoms instead. As the research develops further, many genes and proteins that are related to autophagy and AD pathogenesis have been found, and to elucidate the role of those genes and molecules in the regulation of autophagy for AD pathogenesis is important. Moreover, many recent studies have reported that small molecules modulated autophagy via different regulating pathways in autophagy exerting great benefits in clearing Aβ and Tau, thereby ameliorating AD symptoms. Although autophagy has been extensively studied for its ability to clear Aβ aggregates and phosphorylate Tau protein and protect nerve cells from damage in AD, a deeper and more comprehensive understanding of the role of autophagy in the pathogenesis of AD research is still urgent, which will provide new theories and even therapeutic targets for clinical trials of drug in AD.

## CONFLICT OF INTEREST

The authors declare that they have no competing interests.
